# Floodplain inundation spectrum across the United States

**DOI:** 10.1038/s41467-019-13184-4

**Published:** 2019-11-15

**Authors:** Durelle T. Scott, Jesus D. Gomez-Velez, C. Nathan Jones, Judson W. Harvey

**Affiliations:** 1 Department of Biological Systems Engineering, Virginia Polytechnic Institute and State University, Blacksburg, 24061 VA USA; 2 Department of Civil and Environmental Engineering, Vanderbilt University, Nashville, 37212 TN USA; 3 Department of Biological Sciences, University of Alabama, Tuscaloosa, 35487 AL USA; 4 U.S. Geological Survey, Earth System Processes Division, Reston, 20192 VA USA

**Keywords:** Hydrology, Limnology

## Abstract

Floodplain inundation poses both risks and benefits to society. In this study, we characterize floodplain inundation across the United States using 5800 stream gages. We find that between 4% and 12.6% of a river’s annual flow moves through its floodplains. Flood duration and magnitude is greater in large rivers, whereas the frequency of events is greater in small streams. However, the relative exchange of floodwater between the channel and floodplain is similar across small streams and large rivers, with the exception of the water-limited arid river basins. When summed up across the entire river network, 90% of that exchange occurs in small streams on an annual basis. Our detailed characterization of inundation hydrology provides a unique perspective that the regulatory, management, and research communities can use to help balance both the risks and benefits associated with flooding.

## Abstract (web summary)

The variations in overbank flow from rivers onto floodplains from regional to continental scales are understudied. Here, the authors investigate this variation as a function of hydroclimatic parameters and channel size in the conterminous U.S. and find that the timing of floodplain inundation is largely controlled by regional factors, while the frequency, duration and magnitude of these inundations vary consistently with channel size.

## Introduction

River flooding has posed risks and benefits for early civilizations to modern society. Historically, river flooding replenished fertile floodplain soils in water scarce societies^1^. Urban areas that preferentially occupied river banks are now a locus for catastrophic flood damages, with a potential for increasing flood frequency associated with upstream land uses and effects of climate change that may exacerbate flooding^2,3^. These risks are being increasingly well defined by regional to continental-scale water balance models and flood risk assessments^4,5^, as well as recent efforts that incorporate hydrology into global climate simulations^6,7^. However, moderate floodplain inundation performs services relevant to society ranging from filtering pollutants^8–11^ and flood attenuation^12^ to food web support and habitat for healthy fisheries^13^.

Studies of a broad range of floodplain inundation, from flood initiation when water first enters a floodplain through recession, are needed to understand the role of floodplains in storing water, transforming water quality, and supporting ecosystems. A large obstacle in exploring floodplain inundation throughout river basins has been the ability to quantify when stream water exchanges with the adjacent floodplain. Rather than quantifying floodplain inundation directly, thresholds for floodplain inundation are often estimated by a given return period for maximum flows (e.g. 1.5 year)^14^ based on the inferred significance of bankfull flow in shaping the channel and the frequency analysis of annual maximum flows^15^. In addition, most previous studies only resolve flooding at a daily timescale, which substantially misrepresents short-lived floods in small streams. As a result, previous regional to continental-scale investigations have not fully characterized floodplain inundation^16^.

This study represents one of the first continental-scale investigations examining all floods from the onset to end of inundation across a long-term record. We analyzed sub-hourly flow data from 5800 USGS gaging stations across the conterminous U.S. (https://waterdata.usgs.gov/nwis) and quantified all floodplain inundation events and their characteristic metrics (magnitude, frequency, duration, and seasonal timing) as they vary with river size and hydroclimactic region over a 10-year flow record. First, we estimated a threshold for floodplain inundation and its uncertainty at each gage station using a breakpoint analysis of each gage’s stream gaging measurements—our analysis differs in method but is consistent with quantifying bankfull flow, a widely used metric in fluvial geomorphology^17^. Second, we estimated the floodplain fraction of total river flow, the timing, and duration for each event over the 10-year flow record. As explained in the methods and the SI, we propagated the uncertainty from the breakpoint analysis to bound these metrics. Third, we extrapolated results across the U.S. stream network by building predictive relationships between floodplain inundation metrics and drainage area and compiling metrics by major river basin and stream order (1st–7th order).

We found that floodplains convey a substantial proportion (approximately 4–12.6%) of a river’s annual flow. The median duration of floodplain inundation increased with river size, from less than a day in the smallest streams to many days in the largest rivers. Floodplain event inundation volumes are greater and floods last longer in large rivers, but flooding occurs much more often in small streams; as a result, large rivers and small streams individually account for similar magnitudes of river-floodplain exchange (except in the arid west where exchange decreases as stream order increases). Furthermore, more of the river’s total annual flow interacts with floodplains in the smaller streams compared with larger rivers. A cumulative estimate of river-floodplain exchange suggested that greater than 90% of the river-floodplain exchange occurs in small streams that dominate the cumulative stream length within basins. An improved quantification of inundation hydrology will be useful to isolate where in the network the water-quality functions of floodplains are most important, in order to inform regulatory strategies and help to prioritize management practices that protect water resources and aquatic health of river corridors.

## Results

### Magnitude of floodplain inundation

**Fig. 1:**
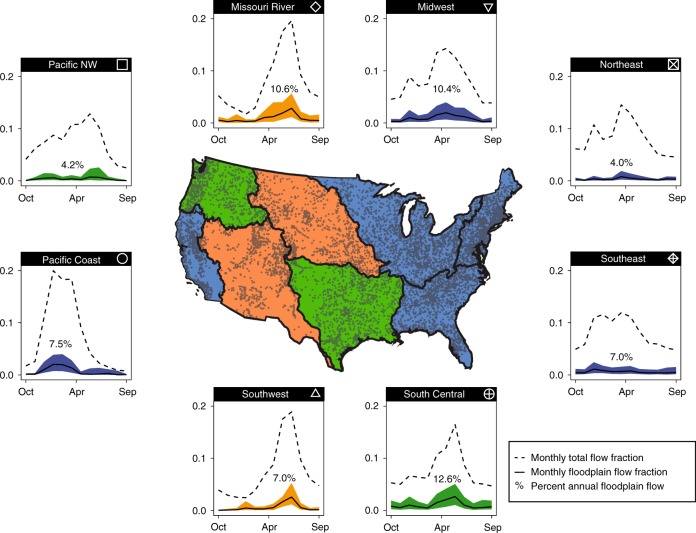
Timing of annual stream flow and inundation. (outside) Regional variation across major river basins of the mean monthly proportion of total annual river flow (dotted line) and mean monthly proportion of floodplain flow to total annual river flow. The highlighted number for each basin represents the percent of total annual river flow on floodplains. The shading represents the 25th and 75th quantiles based on analysis of all gage stations within each basin. (inside) Gage stations and basin boundaries (Northeast; Southeast; Midwest; South Central; Missouri; Southwest; Pacific Northwest; Pacific Coast) are displayed, and the background color (blue, orange, and green) indicates the hydroclimatic signature (rain dominated = blue; snow dominated = orange; and mixed = green)

The median annual magnitude of floodplain inundation ranged between 4.0 and 12.6% of the total annual river flow in eight major river basins (Fig. 1). The proportion of total river flow occurring on floodplains is therefore substantial, especially when compared with the median duration of floodplain inundation for U.S. stream gages, indicating that floodplains are inundated on average for only 2.6% of the year (Supplementary Fig. 1). The magnitude of floodplain inundation varied greater in some basins more than others, ranging from less than 2% within the Northeast and Pacific Northwest basins to over 29% in the South Central basin, as expressed by the difference between the 75th and 25th quantiles in each river basin (Fig. 1).

### Seasonal timing of floodplain inundation

Regional differences in the timing of floodplain inundation are evident across river basins. In the Missouri and Southwest basins (Fig. 1), 56% and 69% of the annual floodplain inundation occurs between April and June, respectively. In contrast, over 71% of the annual floodplain inundation occurs between January and March in the Pacific Coast basin. For the Midwest and South Central basins, a high degree of seasonality in floodplain inundation is shown where 48% and 49% of the annual inundation occurs between March and May. Timing of floodplain inundation is influenced by three important hydroclimatic characteristics: annual snowmelt, balance of snowmelt/rain-on-snow contributions versus rainfall, and proportion of high-intensity rainfall (Fig. 1). These large scale hydroclimatic variables are important drivers that influence the frequency and timing of floodplain inundation^18^. There is a prevalence of snowmelt derived flooding in the spring and early summer in basins such as the Missouri and Southwest basins, compared with the Pacific coast basin where monsoonal driven flooding occurs during the fall and early winter. In the Midwest and South Central basins, a combination of snowmelt, rain on snow, and spring rain events on saturated soils when evaporation is low contribute to the seasonality in the central U.S. Our hydroclimatic interpretations are generally consistent with earlier flood studies that generally separate mechanisms of extreme floods into snowmelt, rain-on snow, and precipitation based events^19,20^ and often highlighted strong gradients in these mechanisms at the continental scale^20,21^.

### Event inundation metrics

**Fig. 2:**
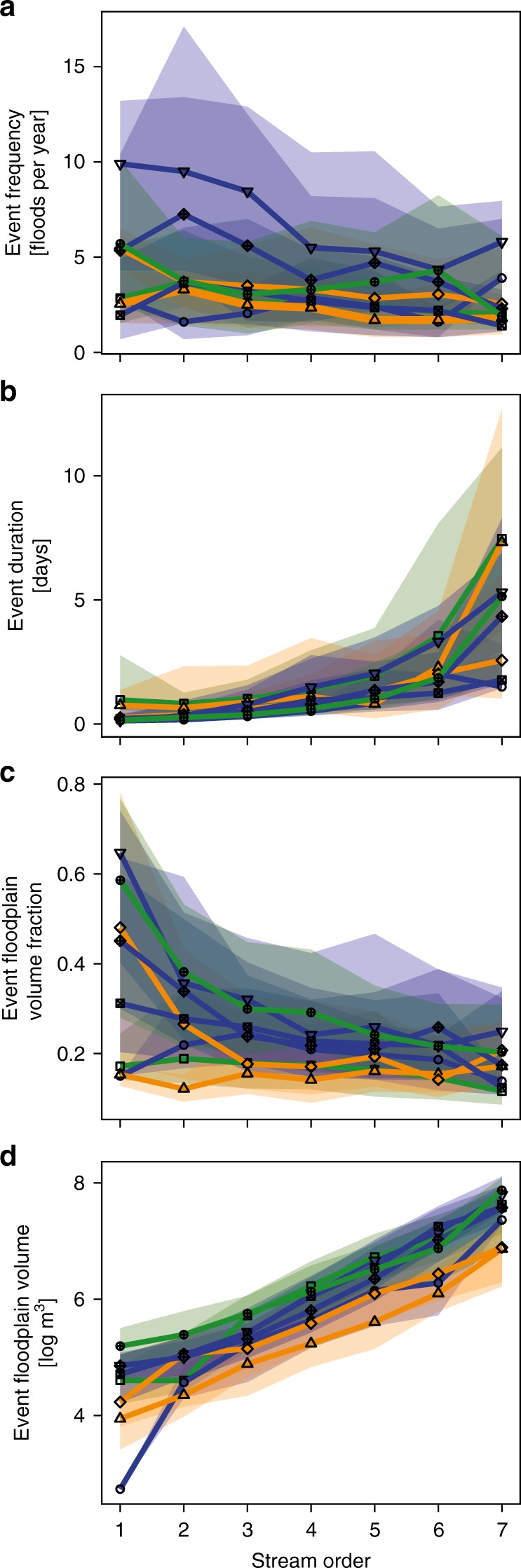
Flood characteristics as a function of stream size. Median (**a**) event frequency [floods year^−1^], (**b**) event duration [days], (**c**) fraction of flood that inundates floodplain, and (**d**) event floodplain inundation volume [log$\,\text{10}\,$ m^3^]. The shading represents the 25th and 75th quartiles of the stations within each major river basin of a given stream size (small—1st order to large—8th order)

We found that the flood event frequency, duration, fraction of flood that inundates floodplain, and the event inundation volumes varied systematically with river size across the U.S. The smallest streams flood more often (Fig. 2a) with a median frequency of four inundation events per year across the U.S. Event frequency in 1st order streams varied by basins, ranging from less than two events per year in the Northeast to over 10 in the Midwest. In contrast, 7th order rivers had a median event frequency of two events per year, but ranged in frequency from one event per year in the Northeast to six events per year in the Midwest. While frequency of flood events generally decreased as a function of stream size across the U.S., the median duration of flooding increased from 5 h to 4.7 days as stream size increased from 1st order streams to 7th order rivers (Fig. 2b). Our results also show that event fractions of floods that inundate floodplains, which represents the fraction of the event’s total river flow that inundates the floodplain, generally decreased with river size (Fig. 2c), from a median of 65% in small streams in the Midwest to less than 12% in larger rivers in the Northeast. The exception was in the western U.S. (Pacific Northwest, Pacific Coast, and Southwest), where the event floodplain inundation volumes averaged 17% of total river flow across all stream orders. While the fraction of floods that inundate the floodplain decreased with river size, the event inundation volumes increased three orders of magnitude from 1st order streams to 7th order rivers across all river basins (Fig. 2d).

### Reach-scale and cumulative river-floodplain exchange

**Fig. 3:**
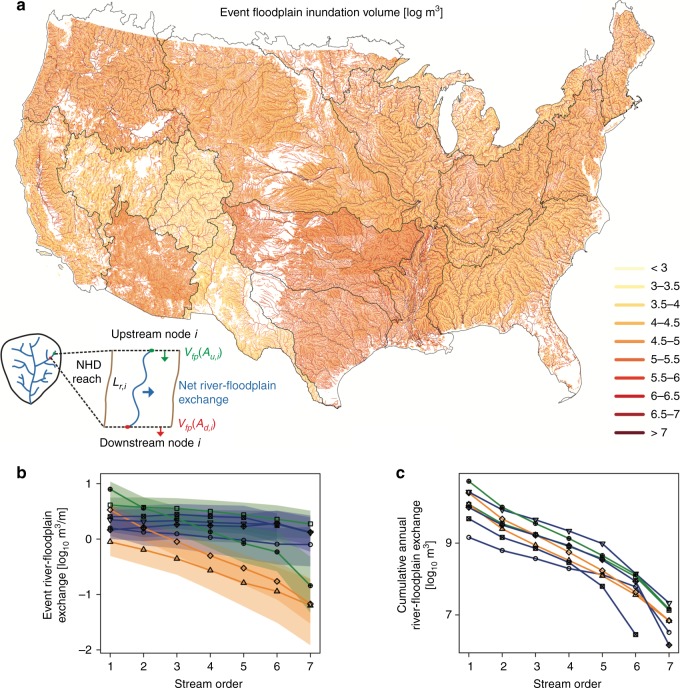
Floodplain significance through river basins applied to the National Hydrography Database Plus stream network. **a** Event floodplain inundation volume [log m^3^], with inset highlighting approach to estimate river-floodplain exchange per reach. **b** River-floodplain exchange during events [log$\,\text{10}\,$ m^3^ m^−1^] within all streams of order *n* across major river basin. The uncertainty bounds represent 95th confidence derived from the uncertainty analysis. **c** Cumulative annual river-floodplain exchange [log$\,\text{10}\,$ m^3^] within each stream order across major river basin

Using the estimates from the gage observational network, we fitted power-law relationships between drainage area and the key descriptors of inundation (e.g., inundation volume and duration at the event and annual time scales), obtaining a parsimonious tool to map these variables into the NHD Plus river network and to get a better perspective of their spatial variability. Figure 3a illustrates this map for the case of floodplain inundation volume during typical events. From this mapped variable, we then calculated event river-floodplain exchange (Fig. 3b) at each NHD reach and also estimated the cumulative river-floodplain exchange throughout all river reaches of each major river basin (Fig. 3c). Event river-floodplain exchange is the difference in floodplain inundation volume between the upstream and downstream reach (Fig. 3a).

Event river-floodplain exchange distinguished two groups, i.e., basins where exchange was constant with stream size and basins where exchange decreased with stream size. Within the snowmelt basins of the arid west (Southwest, Missouri) and the South Central basins, river-floodplain exchange decreased as a function of stream size (Fig. 3b) from a median of 0.9 m^3^ m^−1^ event^−1^ in 1st order streams to a median of 0.1 m^3^ m^−1^ event^−1^ in 7th order streams. In contrast, event river-floodplain exchange within the remaining basins (Northeast, Southeast, Midwest, Pacific Coastal, and Pacific Northwest) was higher and generally less variable, ranging from 4.1 m^3^ m^−1^ event^−1^ in 1st order streams to 1.9 m^3^ m^−1^ event^−1^ in 7th order streams. Across all basins, cumulative annual river-floodplain exchange decreased over 5-orders of magnitude throughout the river network (Fig. 3c), as controlled by the dendritic nature of river networks^22^ and its associated exponential decrease in total river length in each stream order. Although our simple scaling with drainage area to estimate cumulative effects does not explicitly account for detailed spatial factors such as reach-to-reach geomorphic heterogeneity along the river corridor^23^, including valley morphology^24^, channelization^25^, reservoirs for water storage, and flood control structures such as levees^26,27^, it does demonstrate the general tendency for flood magnitude, frequency, duration, and river-floodplain exchange to scale with drainage area, which provides a useful first-order assessment of the cumulative effects that can undoubtedly be improved.

### Implications and future outlook

Continental-scale flood studies typically focus on large, catastrophic flood events. Our study focused on a broad range of flood inundation, from small to large floods and from the initiation of flood inundation through recession. We found that the duration of flooding is generally less than 2.6% of the year across the conterminous U.S., and varied from $<$1% in 1st order streams to over 10% in the larger rivers (Supplementary Fig. 1). Furthermore, the proportion of total river flow reaching floodplains was determined to be a substantial proportion (3–12% median, as high as 29% in the 75th upper quartile, Fig. 1) of the annual river water discharge across the U.S., providing important functions and services at regional to continental scales.

The seasonality of inundation has implications for food production, water storage, urban infrastructure, water quality, sediment storage and supply, and ecological functioning. For example, spring flooding within the South Central and Midwest can delay planting or destroy young crops^28^, but may also recharge groundwater to offset the effects of extreme droughts that decrease crop yields^29^. Flood events during the spring within larger rivers draining into the Gulf of Mexico could partially offset riverine nutrient loads from upstream, given that spring-time nutrient loads are linked to the extent of the hypoxic zone within the Gulf of Mexico^30^. The majority of the other major river basins experience strong seasonal patterns resulting in periods of both high and low floodplain inundation (Fig. 1). For example, in coastal areas of the Pacific Northwest and Pacific Coast river basins, flooding triggers ecologically and culturally significant fall/winter chinook salmon migrations. Not only do the high flow events provide passage to headwater streams for spawning, nutrient-rich floodplain ecosystems provide key habitat for juvenile development and biomass production^31,32^.

The regional variation observed in our flood inundation metrics is attributable to a combination of hydroclimate, geomorphology, and hydrologic alteration across the major river basins. In the arid west (Missouri and Southwest), much of the stream flow is captured within reservoirs for agriculture and human water demands; previous estimates within the Colorado and Rio Grande basins are 100% capture^33^. The influence of water storage and capture is best observed for these two basins and the South Central (which contains the Rio Grande), where river-floodplain exchange decreased as a function of stream size. The Southwest, which has the lowest exchange across all basins also corresponds to the basin that has the lowest precipitation and is the most water stressed^34^. While ultimately much of the water is captured, these basins experience predictable, annual flooding from snowmelt resulting in prolonged flood periods during the late spring. The remaining basins all had relatively uniform river-floodplain exchange and similar event floodplain magnitudes; however, flood events were shorter and less frequent in the Northeast and Pacific Coast. These two basins were characterized by higher floodplain inundation thresholds, which is generally consistent with previously estimated bankfull discharge thresholds from regional analyses of hydraulic geometry^35^; additionally these two regions also have a high degree of hydrologic alteration which could raise or lower floodplain inundation thresholds depending on whether channel incision occurred^36^. Row-crop agriculture can also increase runoff from rainfall^37^, which will impact flood characteristics in agriculturally dominated regions (e.g., Midwest, Southeast). We also found that the Midwest, Southeast, and the Pacific Northwest had higher cumulative river-floodplain exchange throughout the river network, which has implications for water resources management. Positive implications of higher exchange include enhanced habitat, peak-flow reduction, and water quality improvements, but holistic management needs to consider that higher exchange has the potential to increase the delivery of non-point source runoff to freshwater systems.

The major societal costs of catastrophic floods, and the focus of many studies on extreme floods, may leave the impression that river flooding is always detrimental. Recently there has been an emphasis on understanding how moderate flooding, along with other processes such as hyporheic exchange, may influence water-quality and ecological functions of large river networks^11,38–40^. Our results support the need to quantify floodplain inundation throughout the nation’s river corridors to isolate dominant processes, e.g., where and when floodplains dominate the removal of river nutrients, compared with streambed processing during low flow, and its downstream effects on nutrient loading to estuaries. Specifically, the magnitude, duration, and timing of floods affects the contact time of waters on floodplains with reactive floodplain soils^41,42^, which was hypothesized to be a key rate limiting step for biogeochemical reactions and removal efficiencies of riverborne contaminants^11,43^. Field studies have revealed important processes. For example, as flooding begins, floodplain soils and vegetation tend to release nutrients into floodwater which may be a source of nutrients to the river^44^. Following the initial flush, floodplains generally retain nutrients through physical (settling) and biogeochemical (e.g., denitrification) processes that are facilitated by longer contact times with sediment. Thus, flood durations of less than 1 day have generally been found to increase downstream nutrient export as material is flushed from the floodplain^45,46^, whereas the longer duration floods have sufficient times for settling of suspended particles and contact times with floodplain sediments that promote reactions that remove nutrients, e.g., denitrification^8,47^. Consequently, flooding in small (low-order) streams is likely to increase nutrient loading to rivers, especially where flooding is of short duration (Fig. 2b) and where there is high cumulative water storage on floodplains (Fig. 3b). In fact, small streams (orders 1–3) with flood durations less than 1 day dominate water storage on floodplains, cumulatively representing 92% of the total water storage throughout major river basins. Larger rivers, especially in the Midwest, South Central, and Southeast major river basins are potential hotspots for nutrient storage and biogeochemical processing due to their longer durations of river-floodplain exchange that provide ample residence time for nutrient removal (Fig. 2b). The co-occurrence of high nutrient loading within the agricultural Midwest landscape and higher river-floodplain exchange, for example, present an opportunity for targeted enhancement of floodplain restoration in this region. However, cumulative water storage in floodplains adjacent to larger rivers was less than 8% across all major river basins, which limits their overall role in nutrient removal from rivers, especially where constructed levees limit water exchange and material processing^48,49^. More research is needed to better understand river-floodplain exchange, and its impact on downstream fate and transport of materials across scales ranging from individual river reaches to larger river networks. While modeling^16,24,50^ and experimental^51,52^ investigations are beginning to characterize hydrologic exchange flows between rivers and adjacent floodplains, empirical observations are needed to ground those characterizations in reality.

Sustainable river basin management requires balancing competing water demands (e.g., human, ecosystem) combined with both local (reach-scale) and cumulative effects for downstream water storage, flood risk, and water quality functions and river health. Both scientific forefronts and applications by water resource managers can be aided by our broad characterization of floodplain inundation from small streams to large rivers and across hydroclimatic regions of the U.S. Future efforts to maintain, enhance, or reconnect floodplains need to account for the regional differences in the seasonal timing and magnitude of floodplain connectivity, in addition to the effects of short versus longer duration flood events that alter whether the floodplain acts as a nutrient source or sink and cumulative flood attenuation.

## Methods

### General approach

Our inundation analysis consists of four main steps. First, we identified a flooding threshold using a breakpoint analysis and estimate its uncertainty. To this end, we used sub-hourly flow data, stage-discharge ($s-Q$) field measurements and rating curves ($Q={f}_{{\rm{rc}}}(s)$) from 5800 USGS gaging stations (USGS NWIS, http://waterdata.usgs.gov) in the GAGESII database^53^ (see Supplementary Note 1). Second, we identified individual inundation events above the threshold and applied the modified divided channel method (DCM) to separate flood flow into channel and floodplain components. Third, we estimated the following metrics for each flood event across the record: channel volume (${V}_{{\rm{c}}}$), floodplain volume (${V}_{{\rm{fp}}}^{e}$), event duration (${d}_{{\rm{f}}}$), and time between individual events (${b}_{{\rm{f}}}$) (a full list of variables and their definition is available in Supplementary Table 1). The uncertainty in the flooding threshold is propagated through the metric estimation process. Lastly, we generalized our inundation metrics to subdivisions of Major River Basins and stream order ($\omega$) across the stream network using the National Hydrography Database [NHD Plus V2, http://nhd.usgs.gov] (see Supplementary Note 2) by applying regional power-law relationships based on drainage area. This approach allowed us to estimate event reach-scale and cumulative annual floodplain exchange throughout the river network.

### Breakpoint analysis for inundation

For each gage, we established the threshold for floodplain inundation through a segmented regression analysis^54^ of the stage ($s$) and discharge ($Q$) measurements used to define the gage’s rating curve ($Q={f}_{{\rm{rc}}}(s)$). This approach, allowed us to identify breakpoints in the *s*–*Q* relationship as well as their statistical significance and confidence intervals within a rigorous statistical inference framework^55^. The breakpoint between regression segments often has physical meaning^56^, and in this application, we assume it represents the point of incipient flooding, or the minimum flow where river-floodplain inundation occurs (${Q}_{{\rm{bkf}}}$). This is consistent with hydrogeomorphic theory suggesting that there is an abrupt change in cross sectional hydraulic geometry when stream flow transitions from channel flow to overbank flooding^17,57^. Uncertainty associated with this estimate of ${Q}_{{\rm{bkf}}}$ is largely associated with the hysteretic nature of flood flows and river stage^41,58^, flow measurement error^59^, and non-stationarity in stream/floodplain bathymetry^60^. While possibly unsuitable for site level engineering applications (e.g., sizing a channel for balanced sediment transport), we suggest our estimates of the threshold for floodplain inundation stage (${\hat{s}}_{{\rm{bkf}}}$) and discharge (${\hat{Q}}_{{\rm{bkf}}}$) and their confidence intervals (CIs) provide a useful characterization of floodplain inundation to reveal patterns at the continental scale.

For each gaging site, a linear segmented model of the form

1 $$Q(s)={\beta }_{0}+{\beta }_{1}\ s+{\beta }_{2}\ (s-{\psi }_{{\rm{b}}})\ I(s\ {> }\ {\psi }_{{\rm{b}}})$$

was iteratively fitted to find one or multiple breakpoints ${\psi }_{{\rm{b}}}$ and their statistical significance. In Eq. (1), ${\psi }_{{\rm{b}}}$ is the breakpoint and $I(\cdot )$ is the indicator function equal to one when the argument is true. To this end, we used the package segmented implemented in the R statistical software^61^—details of the theoretical and iterative approaches used by segmented can be found in the package’s documentation and the reference^62^.

For each gage station, we tested the models with zero, one, two, and three breakpoints and used the Bayesian information criterion (BIC) to select the most parsimonious model^62^. Based on the BIC, all of the stations were characterized by one or two breakpoints, and in the cases where two breakpoints were identified, the breakpoint with the highest stage value was physically reasonable and had a better agreement with field observations.

The majority of the gage stations resulted in breakpoint-models with high adjusted *R*^2^ fits (Supplementary Fig. 2). The 25th percentile adjusted *R*^2^ was 0.75, the 50th was 0.89, and the 75th was 0.96. Less than 5% of our gages had adjusted *R*^2^ values of less than 0.40. Examples at each of the 25th, 50th, and 75th are provided in Supplementary Fig. 3. To validate the breakpoint analysis, we compared best-fit estimates of ${\hat{Q}}_{{\rm{bkf}}}$ to measured bankfull at 537 gages from across the US. Measured bankfull values were obtained from 36 different regional curve studies across 32 state^63^. Results suggest strong agreement between estimated and measured bankfull (RMSE = 0.5, see Supplementary Fig. 4).

Once a statistically significant breakpoint (${\hat{s}}_{{\rm{bkf}}}$) and its 95% confidence interval $[{s}_{{\rm{bkf}}}^{-},{s}_{{\rm{bkf}}}^{+}]$ were identified, we used the published USGS stage-discharge rating curve to obtain an estimator for the bankfull discharge ${\hat{Q}}_{{\rm{bkf}}}={f}_{rc}({\hat{s}}_{{\rm{bkf}}})$ and its corresponding 95% confidence interval $[{Q}_{{\rm{bkf}}}^{-},{Q}_{{\rm{bkf}}}^{+}]=[{f}_{rc}({s}_{{\rm{bkf}}}^{-}),{f}_{rc}({s}_{{\rm{bkf}}}^{+})]$. We then addressed the uncertainties in the estimation of ${s}_{{\rm{bkf}}}$ and ${Q}_{{\rm{bkf}}}$ by using a Monte Carlo approach^64,65^, where we assumed that the probability distribution function (pdf) for the bankfull stage ${s}_{{\rm{bkf}}}$ is normally distributed with mean ${\hat{s}}_{{\rm{bkf}}}$ and standard deviation ${\sigma }_{{s}_{{\rm{bkf}}}}=({s}_{{\rm{bkf}}}^{+}-{\hat{s}}_{{\rm{bkf}}})/(\sqrt{2}\ {{\rm{erf}}}^{-1}(2\cdot 0.975-1))$. That is, ${s}_{{\rm{bkf}}} \sim N({\hat{s}}_{{\rm{bkf}}},{\sigma }_{{s}_{{\rm{bkf}}}}^{2})$. Once the pdf for ${s}_{{\rm{bkf}}}$ was defined, we generated random realizations of stage (1000 in our analysis) and mapped them through the USGS stage-discharge rating curve ${f}_{rc}(s)$ to obtain an empirical pdf for bankfull discharge (${Q}_{{\rm{bkf}}}$)^65^. The one thousand realizations of ${Q}_{{\rm{bkf}}}$ allowed us to account for the uncertainty of the flooding metrics, repeating the analysis described below for each realization. In other words, this methodology allowed us to estimate empirical probability distribution functions for bankfull stage and discharge and all the metrics characterizing the temporal evolution of river-floodplain inundation at each station.

Our next step was to apply a Guassian smoothing filter to remove spurious fluctuations of stage and discharge within the time series (Supplementary Fig. 5). In this case, a filtered time series is obtained by using a convolution of the form^66^

2 $${Y}_{f}(t)={\int }_{-\infty }^{\infty }g(t-u)Y(t){\rm{d}}u$$

where $Y(t)$ is the original time series and $g(t)$ is a Gaussian kernel given by

3 $$g(t)=\frac{1}{\sqrt{2\pi }\ \sigma }\exp \left(\frac{{t}^{2}}{2{\sigma }^{2}}\right)$$

with a smoothing parameter $\sigma$.

### Event analysis across flow record

For each realization of ${Q}_{{\rm{bkf}}}$, we identified individual inundation events that exceed the randomly generated bankfull discharge across the gage flow record (10 years) and calculated their associated metrics. We calculated the total volume during each event (${V}_{{\rm{t}}}$) by integrating under the hydrograph with respect to time over the duration of each flood event (Supplementary Fig. 6). The total volume can be conceptualized as the sum of the channel (${V}_{{\rm{c}}}$) and floodplain inundation (${V}_{{\rm{fp}}}^{{\rm{e}}}$) volumes during the event, that is ${V}_{{\rm{t}}}={V}_{{\rm{fp}}}^{e}+{V}_{{\rm{c}}}$ (Supplementary Fig. 7). A modification of the Direct Channel Method (DCM)^67^ was used to estimate ${V}_{{\rm{c}}}$ and ${V}_{{\rm{fp}}}^{{\rm{e}}}$. To find ${V}_{{\rm{c}}}$, it is necessary to further subdivide the channel compartment into the bankfull channel and bankfull excess compartments (Supplementary Fig. 7). ${V}_{{\rm{c}}}$ is the sum of total volume of flow in the bankfull channel (${V}_{{\rm{bkf}}}$) and a bankfull excess compartment that remains associated with the channel (${V}_{{\rm{bfe}}}$):

4 $${V}_{{\rm{c}}}={V}_{{\rm{bkf}}}+{V}_{{\rm{bfe}}}$$

Here, ${V}_{{\rm{bkf}}}$ is calculated by integrating the realization of the bankfull flow (${Q}_{{\rm{bkf}}}$) over the course of the event using

5 $${V}_{{\rm{bkf}}}={\int }_{{t}_{{\rm{start}}}}^{{t}_{{\rm{end}}}}{Q}_{{\rm{bkf}}}\ {\rm{d}}t={Q}_{{\rm{bkf}}}\ ({t}_{{\rm{end}}}-{t}_{{\rm{start}}}).$$

Similarly, ${V}_{{\rm{bfe}}}$ is calculated integrating ${U}_{{\rm{bkf}}}$ across the bankfull excess cross sectional area over the course of the individual storm:

6 $${V}_{{\rm{bfe}}}={\int }_{{t}_{{\rm{start}}}}^{{t}_{{\rm{end}}}}{U}_{{\rm{bkf}}}\ {w}_{{\rm{bkf}}}\ (s-{s}_{{\rm{bkf}}})\ {\rm{d}}t.$$

${U}_{{\rm{bkf}}}$ was assumed to represent average velocity in the bankfull excess compartment. ${U}_{{\rm{bkf}}}$ is estimated using the empirical relationships developed by Bjerklie^68^ with predictors derived form the NHD Plus V2 dataset (see Supplementary Note 3 and Supplementary Fig. 8). Bankfull channel width (${w}_{{\rm{bkf}}}$) and depth (${d}_{{\rm{bkf}}}$) were estimated using the downstream hydraulic geometry relationships developed by Wilkerson et al.^69^. Finally, we calculated the floodplain inundation as ${V}_{{\rm{fp}}}^{e}={V}_{{\rm{t}}}-{V}_{{\rm{c}}}$.

The information from the analysis applied across the flow record was then used to quantify variation in the frequency, duration, and magnitude of river-floodplain connectivity with respect to both major river basin and $\omega$. Here, it is important to highlight that both major river basin and $\omega$ categorical variables only provide coarse representation of the observed variability in event floodplain volumes across gages. Major river basin subdivisions each represent multiple U.S. Geological Survey 2-digit Hydrologic Unit Codes (HUCs), and thus often encompass heterogeneous hydroclimatic regimes. Similarly, while $\omega$ provides a useful categorical variable to describe variation along the river corridor^11,70^, regional variation in river network geometry results in wide variability of stream size within a given stream order.

### Application to the US river network

In order to generalize the river flooding metrics to the U.S. river network, we separated the gaging stations by USGS HUC-2 regions (2-digit hydrologic units of approximately 460,000 km${}^{2}$ on average and fitted models of the form:

7 $${\mathrm{log}}_{10}({V}_{{\rm{fp}}}^{{\rm{e}}})={a}_{{V}_{{\rm{fp,e}}}}+{b}_{{V}_{{\rm{fp,e}}}}{\mathrm{log}}_{10}(A),$$

8 $${\mathrm{log}}_{10}({V}_{{\rm{fp}}}^{a})={a}_{{V}_{{\rm{fp,a}}}}+{b}_{{V}_{{\rm{fp,a}}}}{\mathrm{log}}_{10}(A),$$

where the coefficients ${a}_{{V}_{{\rm{fp}}}}$ and ${b}_{{V}_{{\rm{fp}}}}$ were estimated with a Robust Linear Regression function *rlm* implemented in R statistical software^61^ (red lines in the example for the Ohio River Basin, Supplementary Fig. 9). These models allowed us to map these variables along the reaches of the National Hydrography Dataset (NHD Plus Version 2, http://nhd.usgs.gov).

For each reach $i$ of the NHD Plus Version 2 river network, two metrics are of particular interest: the average floodplain exchange per event and per unit length of channel (${E}_{{\rm{fp,}}i}^{{\rm{e}}}$; [L${}^{3}$Event${}^{-1}$L${}^{-1}$]), and the average floodplain exchange per year and per unit length of channel (${E}_{{\rm{fp,}}i}^{{\rm{a}}}$; [L${}^{3}$T${}^{-1}$L${}^{-1}$]). These metrics were calculated for each reach as follows:

9 $${E}_{{\rm{fp}},i}^{{\rm{e}}}=\frac{{V}_{{\rm{fp}}}^{{\rm{e}}}({A}_{{\rm{d}},i})-{V}_{{\rm{fp}}}^{{\rm{e}}}({A}_{{\rm{u}},i})}{{L}_{{\rm{r}},i}}\,,$$

10 $${E}_{{\rm{fp}},i}^{{\rm{a}}}=\frac{{V}_{{\rm{fp}}}^{{\rm{a}}}({A}_{{\rm{d}},i})-{V}_{{\rm{fp}}}^{{\rm{a}}}({A}_{{\rm{u}},i})}{{L}_{{\rm{r}},i}}\,,$$

where ${A}_{{\rm{d}},i}$ and ${A}_{{\rm{u}},i}$ are the drainage areas of the upstream and downstream nodes of the NHD reach, respectively, ${L}_{{\rm{r}},i}$ is the length of the NHD reach, and the floodplain inundation volumes were calculated with Eqs. (7) and (8).

Finally, using these estimates, we calculated key metrics to represent the cumulative effect of flooding along the river network. We define the cumulative floodplain exchange for all reaches of stream order $\omega$ during a year for each major river basin as

11 $${V}_{{\rm{fp}},\omega }^{{\rm{a}}}=\sum _{\forall \ i\in \omega }{E}_{{\rm{fp}},i}^{{\rm{a}}}\ {L}_{{\rm{r}},i},$$

where ${V}_{{\rm{fp}},\omega }^{{\rm{a}}}$ is the cumulative floodplain exchange for all reaches of stream order $\omega$ within their respective major river basin during a year.

## Supplementary information

Supplementary Information

## Data Availability

Data is publicly available from [10.5281/zenodo.3491240].

## Code availability

Code is publicly available from [https://github.com/gomezvelezlab/floodplainInundationCONUS].
