# Supplementary material for: Floodplain inundation spectrum across the United States
Source: Nat Commun. 2019 Nov 15;10:5194. doi: 10.1038/s41467-019-13184-4 (PMC6858325; doi:10.1038/s41467-019-13184-4)
Supplement: Supplementary file 1 — Supplementary Information [file 41467_2019_13184_MOESM1_ESM.pdf]

Supplementary Information to:

Floodplain inundation spectrum across the United States

Scott *et al.*

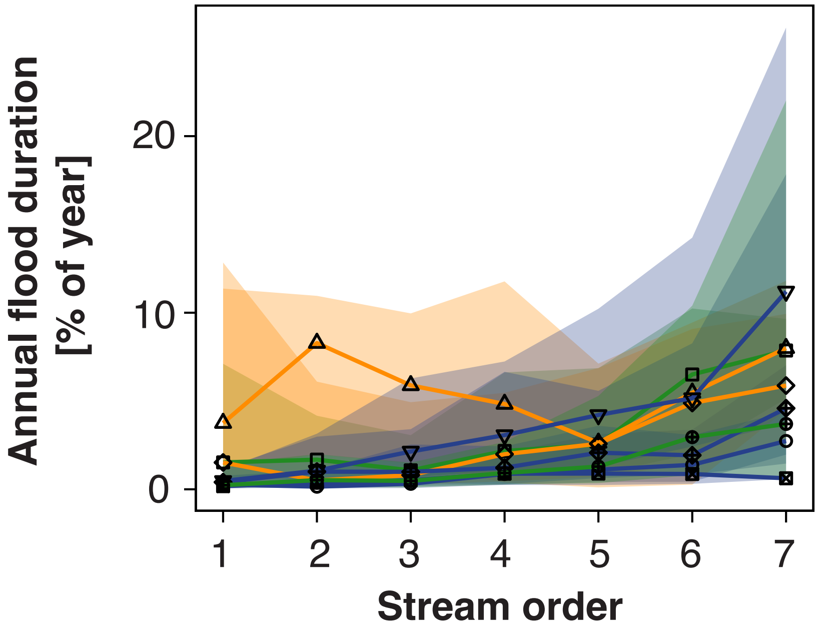

Supplementary Figure 1: Annual flood duration (as a percent of year) for each major river basin as a function of stream order. The shading represents the 25th and 75th quartiles of the stations within each major river basin of a given stream size (small —1st order to large —8th order).

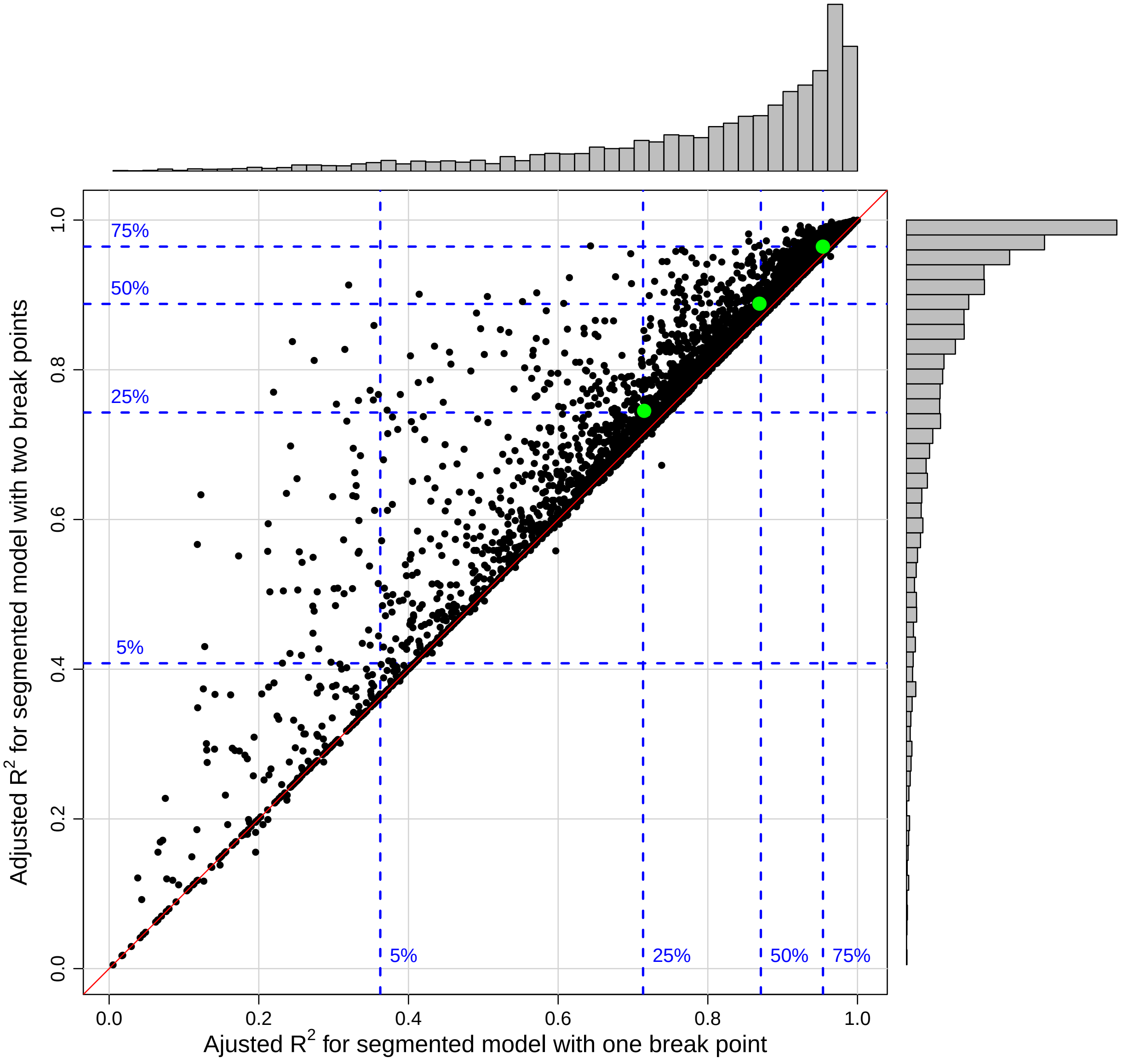

Supplementary Figure 2: Comparison of adjusted R^2 1-break point and 2-break point models for the gages used in our study. The blue-dotted lines represent the percentiles for gages that fall across the 5% to 75% percentiles. The green circles represent the example gages shown in Figure S3. The grey bar plots represent the distrubution of gages with respect to their fit, as measured by R^2.

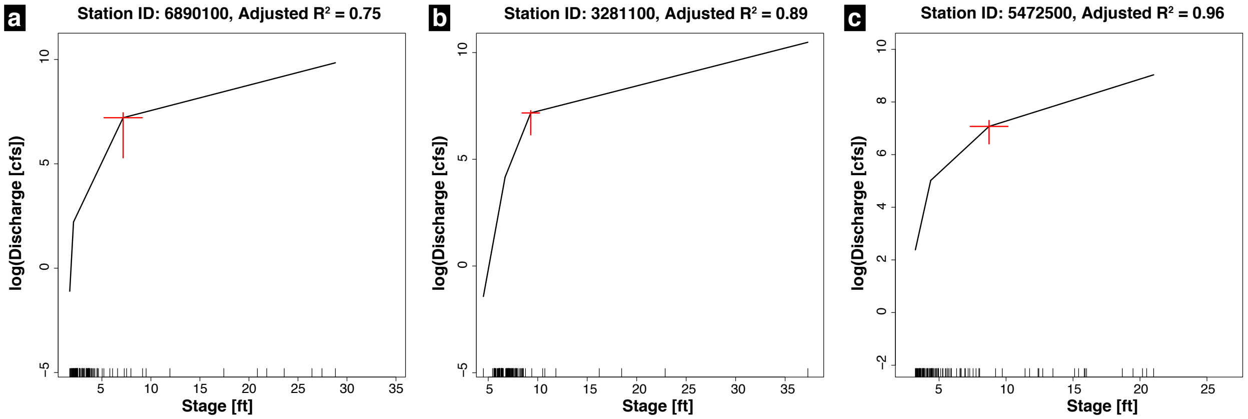

Supplementary Figure 3: Examples from 3 gages for the implemented breakpoint analysis. (a) represents a gage at the 25% percentile, (b) represents a gage at the 50%, and (c) represents a gage at the 75% percentile. The red crosses represent the associated uncertainity for each breakpoint (the min and max are the 25th and 75th percentiles).

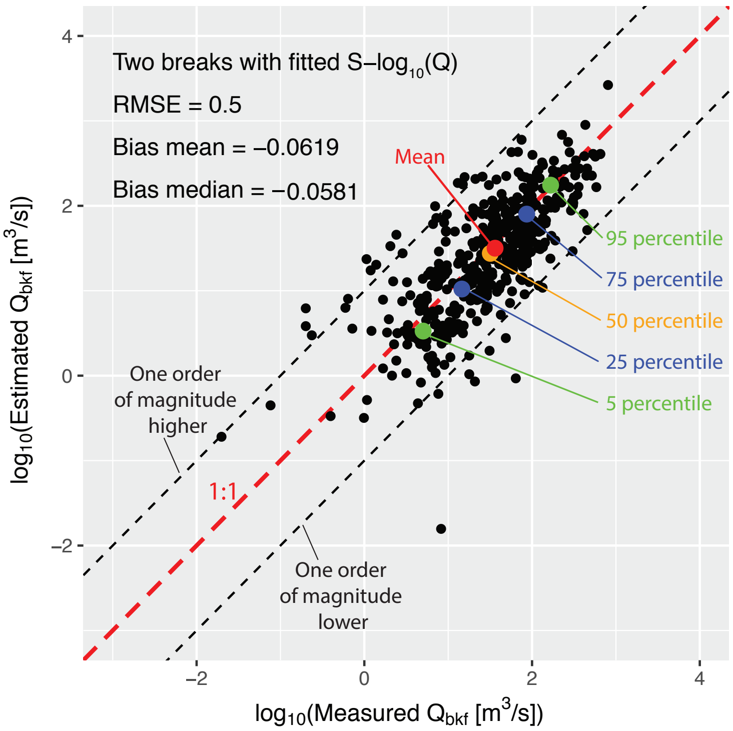

Supplementary Figure 4: Correlation between measured bankfull and modeled breakpoint (Q_{bkf} vs. $\hat{Q}_{bkf}$) at 537 USGS gages across the country. The red dashed line is the 1:1 line, and the lighter dashed lines represent $\pm$ one order of magnitude.

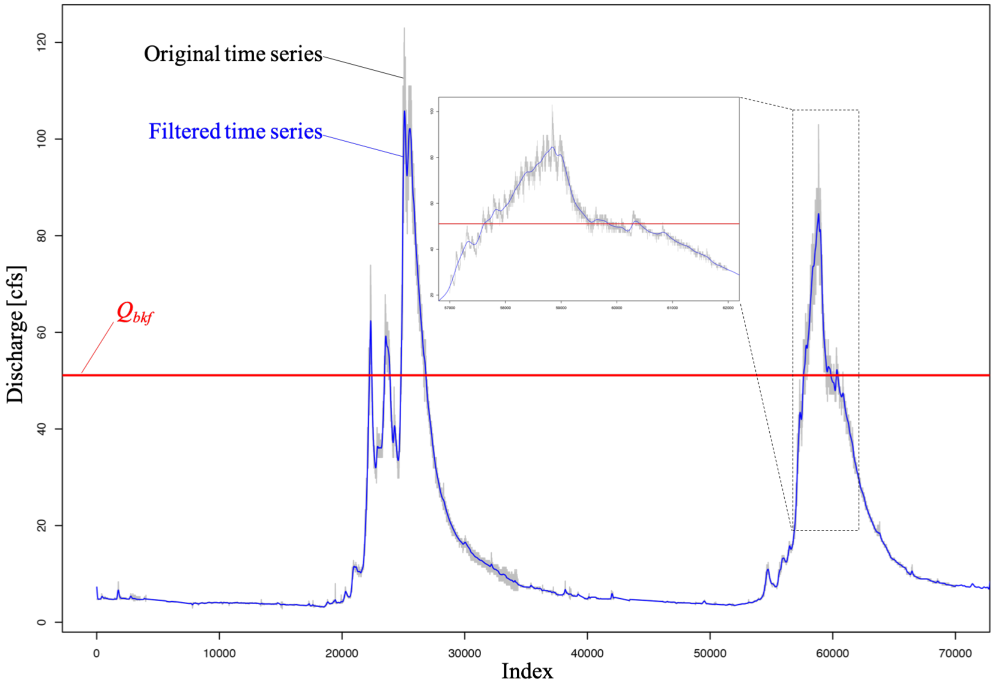

Supplementary Figure 5: Example of an original (grey) and filtered (blue) time series of discharge. In this case, the index corresponds to time increments of 15 minutes. The inset highlights the importance of filtering high-frequency spurious fluctuations in the analysis. Here it was important to select a smoothing parameter σ that removes spurious high-frequency fluctuations while capturing the temporal evolution of the time series. As illustrated by the inset, the presence of spurious fluctuations results in overestimation of events, typically by orders of magnitude. Careful exploration of the approach showed that the skewness and kurtosis of the time series were reasonable indicators to select the magnitude of the parameter σ. In this case, skewness and kurtosis larger than 5 and 50, respectively, were appropriately filtered with a value $\sigma = 1$hr. Otherwise, we used $\sigma = 6$hr. As illustrated in the figure, this results in a well-behaved filter that capture stage and discharge variability.

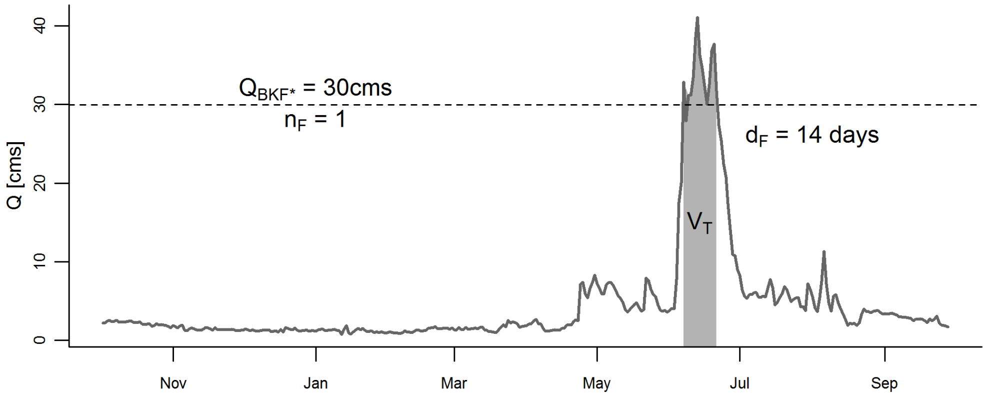

Supplementary Figure 6: Annual hydrograph from Boulder Creek near Boulder, CO across the 1997-1998 water year. As delineated by one realization of Q_{bkf}, the number of floods (n_f) would be equal to 1 for this water year, the duration of that storm (d_f) would be approximately 14 days, and the total flood volume (V_t) would be approximately 41.5×10^6 m^3.

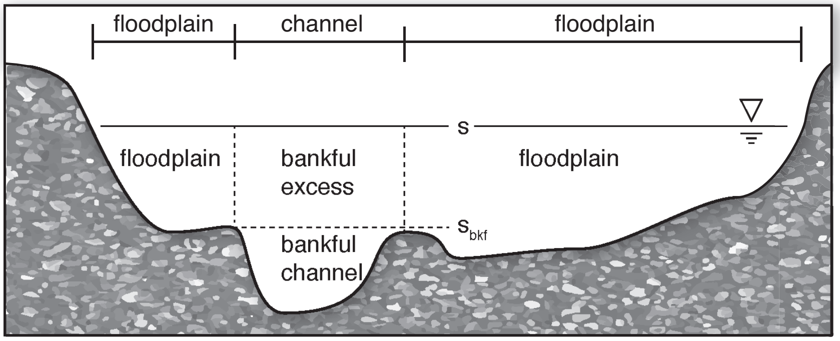

Supplementary Figure 7: Conceptualization of the Divided Channel Method (DCM) where the active river channel cross section is subdivided into floodplain and channel compartments based on topagraphy, river stage (s), and bankful stage (s_{bkf}). The active channel is further divided into bankfull and bankfull excess compartments.

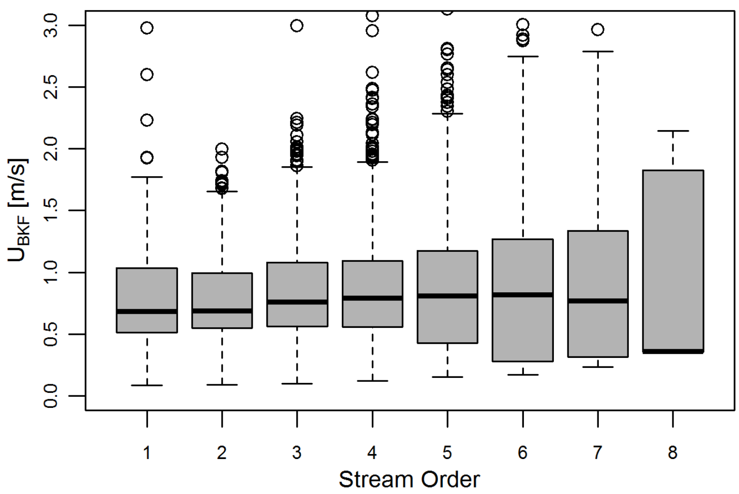

Supplementary Figure 8: Estimate of U_{bkf} at USGS gaging stations across their respective stream orders (ω) using relationship developed by Bjerklie[1].

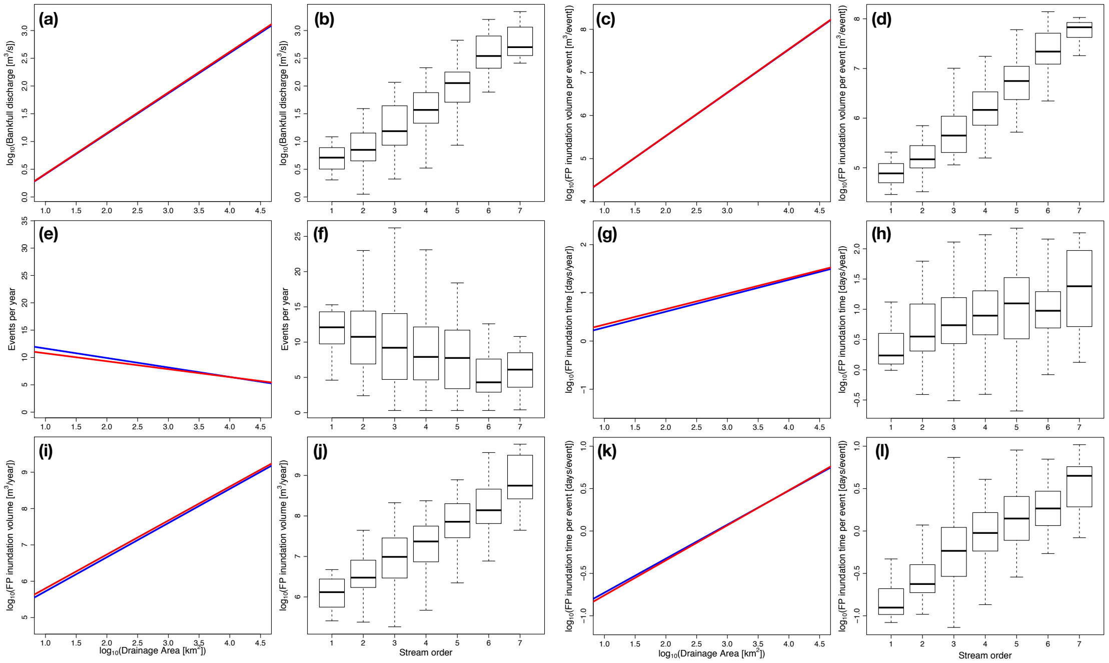

Supplementary Figure 9: Metrics of interest estimated for all gages in the the Ohio River Basin (HUC 05) as a function of drainage area (1st and 3rd columns) and stream order (2nd and 4th columns). (a) and (b) represent bankfull discharge [m^3 s^{-1}], (c) and (d) represent the inundation volume per event [m^3 event^{-1}] (e) and (f) represent the number of events per year, (g) and (h) represent the inundation time per year [days year^{-1}], (i) and (j) represent the floodplain inundation volume per year [m^3 year^{-1}], , and (k) and (l) represent the inundation time per event [days event^{-1}]. Blue and red lines correspond to the linear and robust linear regression fits, respectively.

Supplementary Table 1: Main variables used in analysis

Variable	Description	Units
Q	streamflow	[L^3T^{-1}]
s	stage	[L]
S_c	channel slope	[LL^{-1}]
λ	meander wavelength	[L]
A	contributing catchment area	[L^2]
ω	Strahler stream order	[-]
Q_{bkf}	bankfull discharge	[L^3T^{-1}]
w_{bkf}	bankfull width	[L]
U_{bkf}	bankfull velocity	[LT^{-1}]
	At a station	
n_f	event frequency	[Events Year^{-1}]
d_f	event duration	[T]
D_f	annual duration of inundation	[T]
b_f	time between individual events	[T]
V_t	total volume during event	[L^3]
V_c	channel volume during event	[L^3]
V_{fp}^e	floodplain inundation volume during event	[L^3]
V_{bkf}	bankfull volume during event	[L^3]
V_{bfe}	bankfull excess volume in channel during event	[L^3]
V_{fp}^a	floodplain inundation volume during water year	[L^3]
	For an NHD reach	
L_r	NHD reach length	[L]
E_{fp}^e	event river-floodplain exchange per unit length	[L^3L^{-1}]
T_{fp}^e	event duration	[L^3]
E_{fp}^a	annual river-floodplain exchange per unit length	[L^3L^{-1}]
$V_{fp,\omega}^a$	cumulative floodplain exchange for all reaches of stream order ω during a year	[L^3]

Supplementary Note 1

We used data from both the USGS gaging network [USGS NWIS, `http://waterdata.usgs.gov`] and the NHD Plus geospatial database (NHD Plus V2)[2]. The USGS gaging network consists of over 20,000 gaging stations that continuously collect a variety of measurements describing streamflow, water quality, and various other environmental parameters. We focused our analysis on the gaging stations identified in the GAGESII database ($n = 6,785$)[3]. These sites are located along perennial streams or rivers, have a flow record of at least 10 years, and occur within a watershed that can be reliably delineated. From the GAGESII database, we selected a subset of 5,800 stations with a robust sub-hourly flow record of ten years (October 2007 to September 2017) and with readily available stage-discharge ($s - Q$) field measurements and a corrected stage-discharge relationship (i.e., rating curve, $Q = f_{rc}(s)$). While the majority of stations had 15-minute data, some stations reported 30-minute flow data. Note, sub-hourly flow data is not publicly available prior to 2007 within NWIS.

Supplementary Note 2

The National Hydrography Dataset (NHD Plus Version 2, `http://nhd.usgs.gov`), NHD Plus hereafter, combines data from the National Elevation Dataset (NED) and the National Watershed Boundary Dataset (WBD) into a hydrologically consistent river network for the continental U.S.[4]. The base unit within NHD is the flow line, which represents a relatively short reach of stream or river, with a typical length scale of the order of one kilometer. For each flow line, the database contains information about the individual reach and its contributing watershed (e.g., land use, climate, and annual runoff estimates). For our analysis, we used channel slope (S_c), contributing catchment area (A), and Strahler stream order (ω) from the NHD Plus geodatabase.

Supplementary Note 3

We used empirical relationships to estimate bankfull velocity (U_{bkf}) at each station. In these equations, channel parameters associated with energy regulation such as channel slope (S_{c}) and meander wavelength (λ) serve as independent variables. Unlike other hydraulic geometry variables, drainage area A is not a strong predictor of U_{bkf}[5]. This is because downstream velocity tends to be constant, or slightly increase, as A increases by orders of magnitude[6]. For use in remote sensing applications, Bjerklie[1] proposed two relationships estimating U_{BKF}. The first one highlights the connection between meanders and flow resistance, and similar to Manning's Equation, estimates U_{bkf} as a function of water surface slope, modified meander wavelength (λ), and a fitting factor m that represents an arbitrary fraction of the meander length. The derivation of this theoretical equation provides justification for the second model, an empirical model that relates U_{bkf} with S_{c} and λ:

$$U_{bkf} = 1.37 S_{\mathrm{c}}^{0.31} \lambda^{0.32}. \qquad \text{(Equation1)}$$

Equation (Equation1) is based on geomorphic surveys and flume studies completed by Church and Rood[7] and Leopold et al.[8], respectively. Bjerklie[1] suggest that in the absence of remotely sensed data, wavelength could be estimated using relationship presented in Leopold and Wolman[8] and, more preferable, Williams[9]:

$$\lambda = 10.2 w_{\mathrm{bkf}}^{1.12} \qquad \text{(Equation2)}$$

Supplementary figure 8 displays estimates of U_{bkf} from Equations (Equation1) and (Equation2). Equation (Equation1) is based on a relatively small sample size (n=78); however, the estimates of U_{bkf} are relatively constant across stream orders, which is consistent with general hydrogeomorphic theory (e.g., reference[6]).

Supplementary References

[1] Bjerklie, D. M. Estimating the bankfull velocity and discharge for rivers using remotely sensed river morphology information. *Journal of Hydrology* **341**, 144 – 155 (2007).

[2] U.S. Geological Survey. National hydrography dataset. Available at: https://nhd.usgs.gov/ [Accessed May 6, 2018] (2014).

[3] Falcone, J., Carlisle, D., Wolock, D. & Meador, M. Gages: A stream gage database for evaluating natural and altered flow conditions in the conterminous united states. *Ecology* **91**, 621–621 (2010).

[4] Moore, R. B. & Dewald, T. G. The Road to NHDPlus — Advancements in Digital Stream Networks and Associated Catchments. *JAWRA Journal of the American Water Resources Association* **52**, 890–900 (2016).

[5] Singh, V. P. On the theories of hydraulic geometry. *International journal of sediment research* **18**, 196–218 (2003).

[6] Leopold, L. & Maddock, T. The Hydraulic Geometry of Stream Channels and Some Physiographic Implications. Geological survey professional paper 252, United States Geological Survey (1953).

[7] Church, M. & Belt, K. T. *Catalogue of alluvial river channel regime data* (Dept. of Geography, University of British Columbia, 1983).

[8] Leopold, L. B. & Wolman, M. G. River Meanders. *Geological Society of America Bulletin* **71**, 769 (1960).

[9] Williams, G. P. River meanders and channel size. *Journal of Hydrology* **88**, 147–164 (1986).
